# Ontologies relevant to behaviour change interventions: a method for their development

**DOI:** 10.12688/wellcomeopenres.15908.3

**Published:** 2020-12-18

**Authors:** Alison J. Wright, Emma Norris, Ailbhe N. Finnerty, Marta M. Marques, Marie Johnston, Michael P. Kelly, Janna Hastings, Robert West, Susan Michie

**Affiliations:** 1 Centre for Behaviour Change, University College London, London, UK; 2 ADAPT SFI Research Centre, Trinity College Dublin, Dublin, Ireland; 3 Aberdeen Health Psychology Group, University of Aberdeen, Aberdeen, UK; 4 Primary Care Unit, Institute of Public Health, University of Cambridge, Cambridge, UK; 5 Research Department of Epidemiology & Public Health, University College London, London, UK

**Keywords:** behaviour, behaviour change, ontologies, interventions, evidence synthesis, evaluation studies

## Abstract

**Background:** Behaviour and behaviour change are integral to many aspects of wellbeing and sustainability. However, reporting behaviour change interventions accurately and synthesising evidence about effective interventions is hindered by lacking a shared, scientific terminology to describe intervention characteristics. Ontologies are standardised frameworks that provide controlled vocabularies to help unify and connect scientific fields. To date, there is no published guidance on the specific methods required to develop ontologies relevant to behaviour change. We report the creation and refinement of a method for developing ontologies that make up the Behaviour Change Intervention Ontology (BCIO).

**Aims: **(1) To describe the development method of the BCIO and explain its rationale; (2) To provide guidance on implementing the activities within the development method.

**Method and results: **The method for developing ontologies relevant to behaviour change interventions was constructed by considering principles of good practice in ontology development and identifying key activities required to follow those principles. The method’s details were refined through application to developing two ontologies. The resulting ontology development method involved: (1) defining the ontology’s scope; (2) identifying key entities; (3) refining the ontology through an iterative process of literature annotation, discussion and revision; (4) expert stakeholder review; (5) testing inter-rater reliability; (6) specifying relationships between entities, and; (7) disseminating and maintaining the ontology. Guidance is provided for conducting relevant activities for each step.

**Conclusions:** We have developed a detailed method for creating ontologies relevant to behaviour change interventions, together with practical guidance for each step, reflecting principles of good practice in ontology development. The most novel aspects of the method are the use of formal mechanisms for literature annotation and expert stakeholder review to develop and improve the ontology content. We suggest the mnemonic SELAR3, representing the method’s first six steps as Scope, Entities, Literature Annotation, Review, Reliability, Relationships.

## Introduction

Changing behaviour at individual, community, organisational and population levels is essential to meet the considerable challenges we face in improving population health and wellbeing and environmental sustainability. There is a large, and rapidly growing, body of literature regarding the effectiveness of behaviour change interventions, defined as “interventions that have the aim of influencing human behaviour,” involving the use of products, services, activities, rules or environmental objects ( Michie *et al.*, 2020). Systematic reviews gather and synthesise evidence about these interventions’ effectiveness. However, the volume, complexity and heterogeneity of reported studies are barriers to efficient, timely and useful evidence syntheses. 

Behaviour change interventions can vary greatly in their content and delivery methods, their mechanisms of action, the characteristics of their settings and target populations and behaviours. The lack of shared, scientific terminology across disciplines to describe these characteristics makes it difficult to interpret reports of interventions or to identify and group similar interventions in evidence syntheses. Published studies often include incomplete and inconsistent reporting of interventions, study methods and findings ( Ioannidis *et al.*, 2014), although some improvement has been observed following the publication of reporting guidelines ( Hoffmann *et al.*, 2014; Montgomery *et al.*, 2018; Schulz *et al.*, 2010). To reduce waste in research and maximise the speed of evidence accumulation, there is a need to develop a shared vocabulary for describing key characteristics of behaviour change interventions and for specifying the inter-relationships between those characteristics. Developing an ***ontology*** of behaviour change interventions is an important step towards meeting this need (see glossary of italicised terms, Table 1).

**Table 1.** Glossary of terms used in this article.

Term	Definition	Source
**Annotation**	Process of coding selected parts of documents or other resources to identify the presence of ontology entities	Michie *et al.*, 2017.
**Annotation** **guidance manual**	Written guidance on how to identify and tag pieces of text from intervention evaluation reports with specific codes relating to entities in the ontology.	
**Annotation issues** **log**	Written tracker of problems identified when annotating papers. This included conceptual issues such as study details that did not correspond to ontology classes, and technical issues, such as PDFs not being formatted correctly.	
**Artificial** **intelligence**	The practice of building computer programs to perform tasks that a human would reasonably regard as requiring intelligence.	Nilsson, 2014.
**Basic Formal** **Ontology (BFO)**	An upper level ontology consisting of continuants and occurrents developed to support integration, especially of data obtained through scientific research.	Arp *et al.*, 2015.
**Behaviour change** **intervention (BCI)** **evaluation study**	An intervention evaluation study of a behaviour change intervention scenario	Michie *et al.*, 2020.
**Behaviour change** **intervention (BCI)** **scenario**	A process in which a BCI is applied in a given context, including BCI engagement and outcome behaviour	Michie *et al.*, 2020.
**Coding record**	Report produced by EPPI-Reviewer software used to annotate papers, presenting side-by- side comparison of the coding of two paired annotators.	*EPPI-Reviewer 4 Manual: https://eppi.ioe.ac.uk/cms/Portals/35/* *Manuals/ER4.5.0%20user%20manuala.pdf?ver=2015-10-12-* *122019-620*
**Entity**	Anything that exists, that can be a continuant or an occurrent as defined in the Basic Formal Ontology.	Arp *et al.*, 2015.
**EPPI-Reviewer**	A web-based software program for managing and analysing data in all types of systematic review (meta-analysis, framework synthesis, thematic synthesis etc. It manages references, stores PDF files and facilitates qualitative and quantitative analyses such as meta-analysis and thematic synthesis. It also has a facilitate to annotate published papers.	Thomas *et al.*, 2010. *EPPI-Reviewer 4: http://eppi.ioe.ac.uk/eppireviewer4/* *EPPI-Reviewer Web Version: https://eppi.ioe.ac.uk/eppireviewer-* *web/*
**GitHub**	A web-based platform used as a repository for sharing code, allowing version control.	*https://github.com/*
**Granularity**	Level of detail and specificity required within a given ontology.	Arp *et al.*, 2015.
**Inter-rater reliability**	Statistical assessment of similarity and dissimilarity of coding between two or more coders. If inter-rater reliability is high this suggests that ontology entity definitions and labels are being interpreted similarly by the coders.	Gwet, 2014.
**Interoperability**	Two systems are interoperable if data coming from each system can be used by the other system. Note: An ontology is interoperable with another ontology if it can be used together with or re-uses parts from the other ontology	http://www.obofoundry.org/principles/fp-010-collaboration.html
**Issue tracker**	An online log for problems identified by users accessing and using an ontology.	BCIO Issue Tracker: https://github.com/ HumanBehaviourChangeProject/ontologies/issues
**Minimum** **Information for** **Reporting an** **Ontology (MIRO)** **guideline**	The Minimum Information Required for reporting Ontologies guidelines aiming to facilitate completeness and consistency in ontology documentation and reporting.	Matentzoglu *et al.*, 2018.
**OBO Foundry**	The Open Biological and Biomedical Ontology (OBO) Foundry is a collective of ontology developers that are committed to collaboration and adherence to shared principles. The mission of the OBO Foundry is to develop a family of interoperable ontologies that are both logically well-formed and scientifically accurate. OBO Foundry: *http://www.obofoundry.org/*	Smith *et al.*, 2007.
**OBO Foundry** **principles**	Good practice principles of ontology development and maintenance intended as normative for OBO Foundry ontologies. Ontologies submitted to OBO Foundry are evaluated against them.	http://www.obofoundry.org/principles/fp-000-summary.html
**Ontology**	A standardised framework providing a set of terms that can be used for the consistent annotation (or “tagging”) of data and information across disciplinary and research community boundaries.	Arp *et al.*, 2015.
**Parent class**	A class within an ontology that is hierarchically related to one or more child (subsumed) classes such that all members of the child class are also members of the parent class and all properties of the parent class are also properties of the child class.	Arp *et al.*, 2015.
**Reconciliation**	The process of discussing differences between the annotations of two paired annotators on the same papers. Differences are discussed before a final reconciled version of coding for each paper is produced.	Stan *et al.*, 2014.
**ROBOT**	An automated library and command line tool for ontology workflows.	Jackson *et al.*, 2019, http://robot.obolibrary.org
**URI**	A string of characters that unambiguously identifies an ontology or an individual entity within an ontology. Having URI identifiers is one of the OBO Foundry principles.	http://www.obofoundry.org/principles/fp-003-uris.html
**Versioning**	Ontologies that have been released are expected to change over time as they are developed and refined, leading to a series of different files. Consumers of ontologies must be able to specify exactly which ontology files they used to encode their data or build their applications and be able to retrieve unaltered copies of those files in perpetuity. Versioning is one of the OBO Foundry principles.	http://www.obofoundry.org/principles/fp-004-versioning.html
**Web Ontology** **Language (OWL)**	A formal language for describing ontologies. It provides methods to model classes of “things”, how they relate to each other and the properties they have. OWL is designed to be interpreted by computer programs and is extensively used in the Semantic Web where rich knowledge about web documents and the relationships between them are represented using OWL syntax.	https://www.w3.org/TR/owl2-quick-reference/

Ontologies are classification systems that systematically articulate the inter-relationships between carefully defined “ ***entities***” (phenomena of interest) ( Arp *et al.*. 2015). An ontology provides a set of (1) unique, unambiguous identifiers representing types of entity (including objects, attributes and processes), (2) labels and definitions associated with each of those identifiers and (3) specified relationships between the entities ( Arp *et al.*, 2015). Using an ontology can help integrate data from a variety of disparate research studies and provide a link between different academic disciplines ( Hastings, 2017). 

Ontologies have been developed for numerous scientific domains, including chemistry ( Degtyarenko *et al.*, 2008), biology ( Ashburner *et al.*, 2000), statistics ( Zheng *et al.*, 2016) and biomedical investigations ( Bandrowski *et al.*, 2016). Many ontologies are collected together in the context of the Open Biological and Biomedical Ontology (OBO) Foundry ( Smith *et al.*, 2007). An important foundational good practice principle for the development of scientific ontologies is the methodology of “ontological realism” ( Arp *et al.*, 2015; Smith & Ceusters, 2010), the approach that underpins the Basic Formal Ontology ( Arp *et al.*, 2015, Grenon *et al.*, 2004, Smith & Grenon, 2004). Ontological realism is a methodological framework that emphasizes that the reference for entities that are included in scientific ontologies should be the actual entities in the world, rather than ideas or concepts about those entities in peoples’ minds. Thus, ontological realism can be contrasted with approaches to ontology development that take as their objective the representation of *concepts* ( Smith, 2004). In other words, ontologies should do more than represent knowledge, which necessarily differs from person to person, and instead attempt to represent the world, the world always being consistent with itself. Thereby, the ontology aims to provide an anchor to support the debates and disagreements that may arise in scientific research contexts by ensuring that all parties in a disagreement at least agree on the real-world referents of the entities they are disagreeing about, even if they may disagree about the properties that those entities may hold. Thus, for example, the referent for an ontology entity such as “environmental setting” is the actual environmental entity in the world and not any person or group’s conceptualization of that environmental entity, and the definitions included in the ontology reflect this. Of course, in the social and behavioural sciences, knowledge and other psychological entities are the subjects of research in their own right, and they are perfectly valid entities for inclusion in the ontology, as indeed they are also part of the world.

The ***OBO Foundry*** promotes collaboration and ***interoperability*** of ontologies across scientific domains in several ways, including by providing a common framework for structuring ontologies. This common framework includes a shared understanding of the basic types of entity in the world, implemented as the ***Basic Formal Ontology*** (BFO; Arp *et al.*, 2015; Grenon *et al.*, 2004; Smith & Grenon, 2004). BFO divides entities in the world into two overarching categories: “continuants”, objects and spatial entities that continue to exist over time, such as a geographical setting, and “occurrents”, events or processes, such as the implementation of a behaviour change intervention, that occur or happen in time ( Arp *et al.*, 2015). BFO is a domain neutral ‘top level’ or ‘formal’ ontology, which provides ***parent classes*** beneath which ontologies relating to specific scientific domains can be developed. Having BFO provide a common top-level structure for ontologies facilitates the seamless integration of numerous domain-specific ontologies, creating a situation in which information held in separate repositories can be part of a common framework for categorising and reasoning about the entities in the corresponding domains ( Arp *et al.*, 2015).

Because ontologies define key entities in a field in a format that is accessible to both humans and computers, they can be used to enable the automation of literature searching, ***annotating*** the content of study reports and synthesising findings ( Hastings, 2017). However, at present, no ontology exists that captures the full breadth and detail required to adequately characterise behaviour change interventions ( Norris *et al.*, 2019). The Human Behaviour-Change Project (HBCP) ( Michie *et al.*, 2017) is developing a Behaviour Change Intervention Ontology (BCIO) as part of its goal of developing an automated Knowledge System for evidence synthesis, interpretation and generation. The BCIO will consist of the entities that are key to understanding behaviour change intervention effectiveness as reported in evaluation studies. The HBCP will use the BCIO to annotate behaviour change intervention evaluation reports (henceforth referred to as reports) to train algorithms to automatically extract information from trial reports and feed the data into a ‘Knowledge System’. Algorithms within the knowledge system, developed using ***artificial intelligence*** (AI) approaches will make predictions based on the evidence in response to users’ queries about the most effective interventions in a wide variety of situations (e.g. type of behaviour, mode of delivery, population, setting).

This paper describes the method of ontology development utilised within the HBCP. The papers describing the intervention setting and population ontologies ( Norris *et al.*, 2020b) in this Collection serve as case studies of its application and utility.

### Good practice in ontology development

There are a number of principles of good practice in ontology development that are relevant to building ontologies relevant to behaviour change interventions. Since the BCIO ontologies are being developed for use by the behavioural and broader scientific community, the ***OBO Foundry principles*** have been used as the starting point ( Smith *et al.*, 2007). First, an ontology should have a clearly specified scope and content that is scientifically sound and adheres to that scope. ( http://www.obofoundry.org/principles/fp-005-delineated-content.html). Secondly, the ontology should meet the needs of the relevant community of users ( http://www.obofoundry.org/principles/fp-010-collaboration.html), which for the BCIO includes researchers, policy-makers, planners and practitioners interested in behaviour change interventions. Thirdly, to meet the needs of ontology users, developers should follow naming conventions, such as keeping class names short and memorable, but precise enough to capture the intended meaning ( Schober *et al.*, 2009) and providing appropriate textual definitions for the majority of its classes, enabling humans to understand what qualifies as a member of each ( Seppälä *et al.*, 2017). Fourthly, ontologies should be logically consistent and have clear structures, with the preference being for a well-organised hierarchical structure ( Rudnicki *et al.*, 2016). Fifthly, to avoid duplication of content and maximise the extent to which a collection of ontologies can work seamlessly together, ontology developers should maximise the new ontology’s interoperability with existing ontologies. To achieve this, a new ontology should reuse entities from existing ontologies where appropriate ( http://www.obofoundry.org/principles/fp-010-collaboration.html). Throughout the ontology development process, developers should abide by several core principles. First, perspectivalism. which means admitting that there are multiple true perspectives on the same underlying subject matter and consulting these perspectives while developing the ontology. Secondly, fallibilism, which means acknowledging that “settled” scientific facts can still be subject to update in response to new discoveries and so a method for revising the ontology is required. Finally, open-endedness acknowledges that it should always be possible to add further classes to an ontology in response to user need and therefore an ontology is never “finished”.

### Upper level of the Behaviour Change Intervention Ontology (BCIO) – defining key entities and their scope

**Figure 1.**
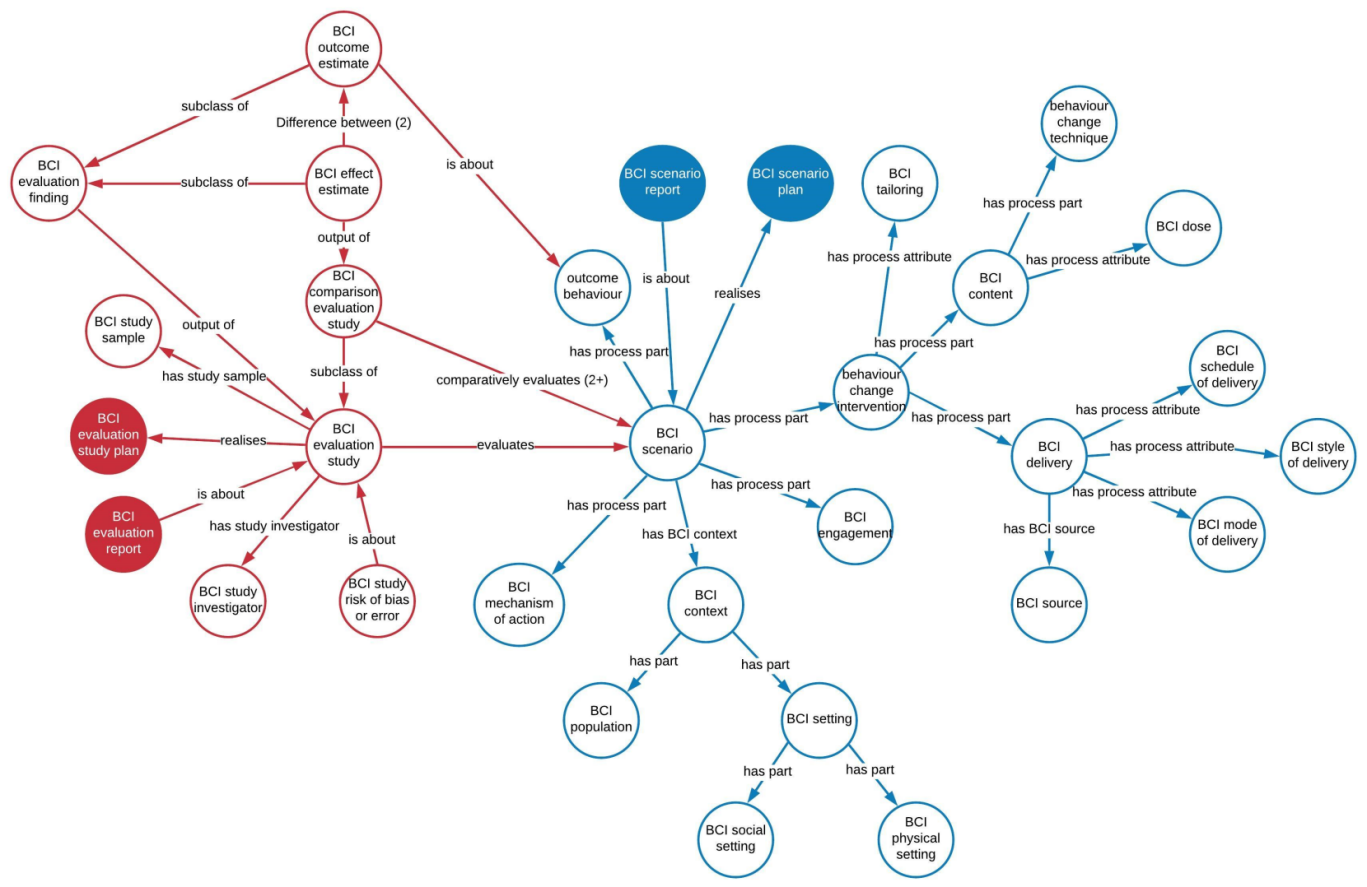
The Behaviour Change Intervention Ontology v1.4 ( Michie *et al.*, 2020).

The upper level of the BCIO ( Michie *et al.*, 2020) comprises key entities relevant to behaviour change interventions and their evaluations and defines the scope of these entities. The upper level is structured according to BFO ( Arp *et al.*, 2015). Use of BFO to structure an ontology is a recommended prerequisite for registration on OBO Foundry ( Smith *et al.*, 2007). The upper level of the BCIO ( Michie *et al.*, 2020) was developed by experts in behaviour change and social science identifying key classes of entity relevant to behaviour change interventions and their evaluations. The initial version of the BCIO was reviewed by a wider group of behavioural science experts and revised. Feedback on the revised version was then sought from three international experts in ontologies, resulting in the current version of the BCIO ( Figure 1). The BCIO’s upper level and the methods for developing it are described in full in a linked paper in this collection ( Michie *et al.*, 2020).

Key entities in the upper level of the BCIO include the Behaviour Change Intervention (BCI) evaluation study and the Behaviour Change Intervention scenario. A Behaviour Change Intervention scenario is formally defined as the “process in which a behaviour change intervention is applied in a given context, including BCI engagement and outcome behaviour,’ ( Michie *et al.*, 2020) in other words the process in which a BCI is applied in a given context (i.e. to a particular population, in a particular setting) and includes engagement with the behaviour change intervention and the nature of the outcome behaviour. Entities relating to the BCI scenario are shown in blue in Figure 1. The BCI evaluation study is formally defined as an ‘intervention evaluation study of a BCI scenario,’ ( Michie *et al.*, 2020) in other words a research study focused on a behaviour change intervention scenario, which has as its output a BCI evaluation finding. Key BCIO entities relating to the BCI evaluation study are shown in red in Figure 1

**Figure 2.**
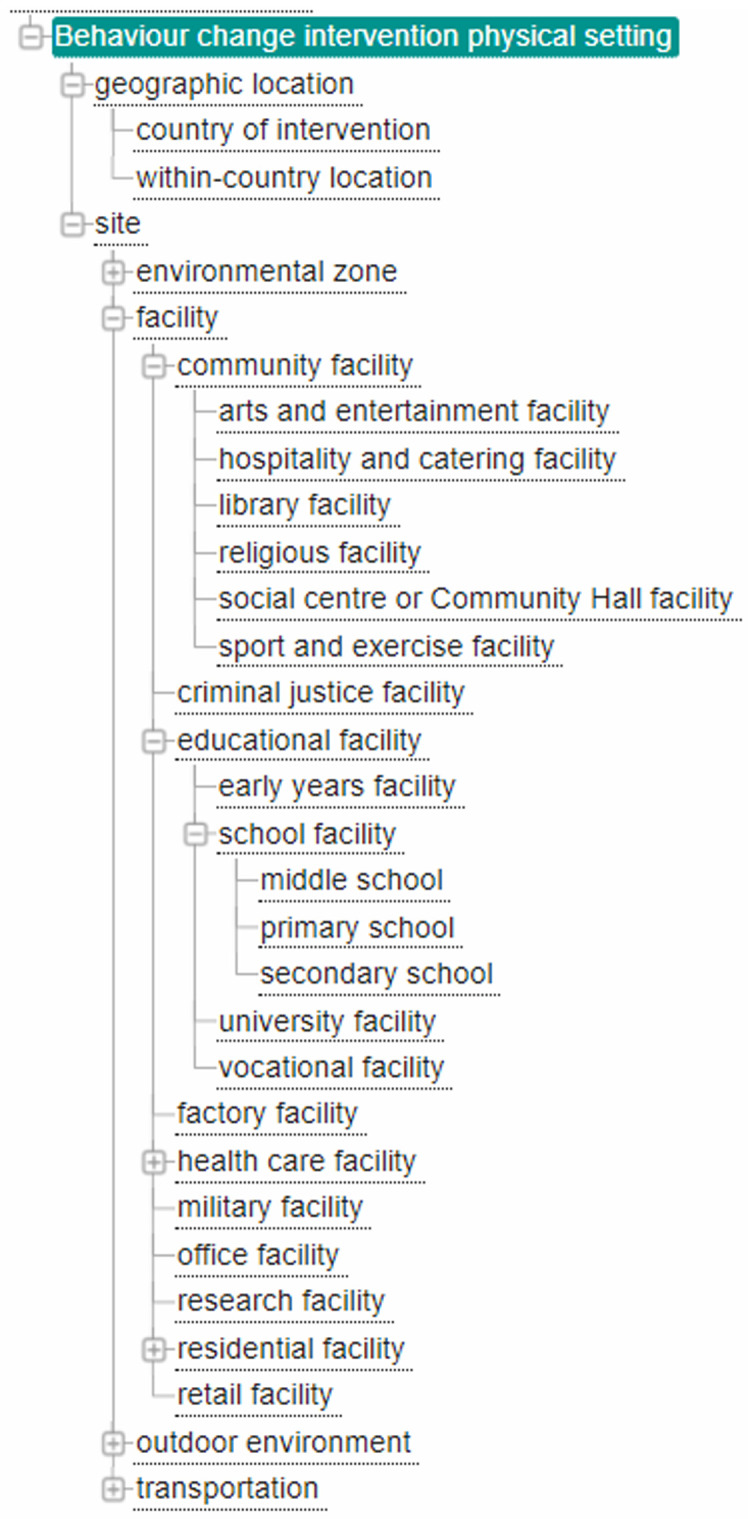
Extract from the Intervention Setting Ontology ( Norris *et al.*, 2020b) showing how lower level entities sit underneath the classes in the upper level of the BCIO (as displayed by the Ontology Lookup Service, https://www.ebi.ac.uk/ols/ontologies/bcio/terms?iri=http://humanbehaviourchange.org/ontology/BCIO_026000) ( Jupp *et al.*, 2015).

In order to represent the complexity of behaviour change interventions with appropriate ***granularity***, most of the upper-level classes in the BCIO will have a large number of subclasses. For example, Figure 2 demonstrates how some of the lower level entities from the intervention setting ontology ( Norris *et al.*, 2020b), sit underneath the BCIO upper level entity of “BCI physical setting”. A method for systematically developing the lower levels of the ontology that lie underneath the key BCIO entities, such as Setting, Population, Engagement, Behaviour, Mode of Delivery, Style of Delivery, Schedule and Source, was required. This paper describes such a method.

### Methods for ontology development

There are a number of methods that can be used to develop ontologies ( Arp *et al.*, 2015; Norris *et al.*, 2019; Noy & McGuinness, 2001). First, existing, non-ontological classification systems, such as taxonomies or terminologies, can be adapted and incorporated. Secondly, developers can search for appropriate entities from existing ontologies to reuse. In addition, developers can employ a range of data-driven approaches to identify classes and relationships. These include annotating ****** published literature (i.e. coding selected parts of documents to identify the presence of ontology entities) and applying the ontology to code datasets. Feedback from potential users can be sought and incorporated at various stages during the ontology development process.

A challenge for ontology developers is to determine how best to sequence and combine methods during development. There is no published guidance that we are aware of on how to develop ontologies relevant to behaviour change and therefore the development team constructed a method *de novo* to create the BCIO. An initial version of the development method for the lower levels of the BCIO was created, based on adhering to the principles of good practice described above and attempting to incorporate the methods mentioned above. We refined and added detail to the method as a result of experiences while developing the Setting and Population ontologies. 

### Aims and objectives

- To describe the development method of the BCIO and explain its rationale;
- To provide guidance on implementing the activities within the development method.

## Methods

The initial version of the ontology development method had seven steps:

1. Development of the scope and definition of the ontology
2. Identifying key entities and developing their preliminary definitions
3. Refining the ontology through an iterative process of literature annotation, discussion and revision
4. Expert stakeholder review
5. Testing inter-rater reliability and making revisions
6. Specifying relationships between entities in the ontology
7. Disseminating and maintaining the ontology

**Figure 3.**
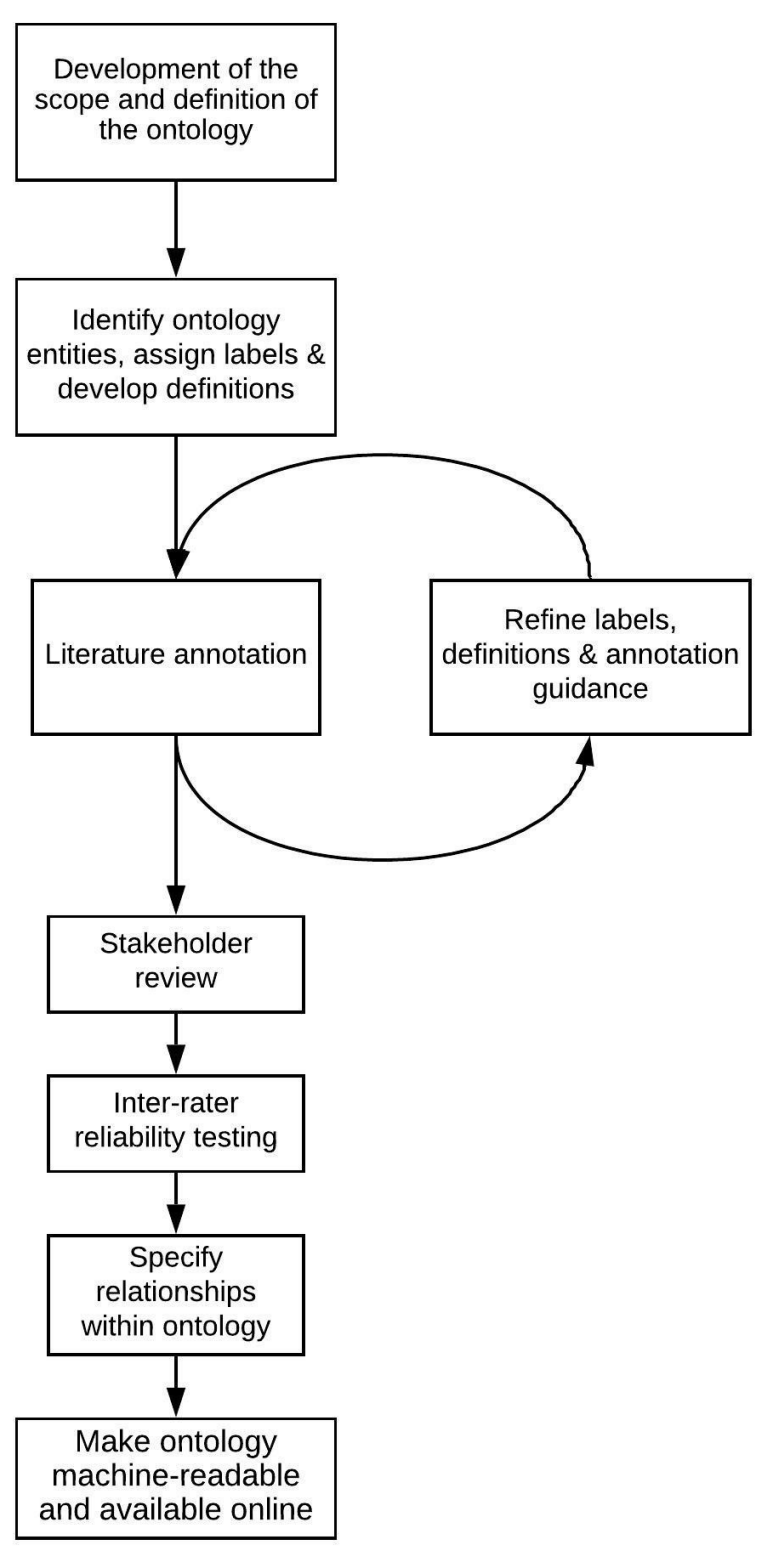
Ontology development method overview diagram.

We discuss the rationale for each step, in relation to good practice in ontology development. We recommend specific activities for each step, together with practical considerations for conducting those activities in a manner most likely to result in an ontology that covers its intended scope and reflects the scientific consensus. The refined, final version of the ontology development method is summarised in Figure 3 and presented in full in Table 2.

**Table 2.** The refined ontology development method.

Step	Relation to good practice in ontology development	Recommended activities	Practical reasons for recommendations
**Step 1: Development of the scope and definition of the ontology**			
a) Define the overall topic of the ontology	Establishes the subject matter the ontology is intended to cover, so ensuring that the ontology will only include relevant content ( http://www.obofoundry.org/ principles/fp-005-delineated- content.html)	Seek relevant sources, such as: • A scoping or other review of how the entity has been operationalised that provides a definition • Previous expert consensus work on the relevant concept • In the absence of the above, consult dictionaries	
**Step 2: Identify key entities and develop their preliminary definitions**			
a) Data driven scoping of relevant entities	Provides a data-driven basis for selecting entities for inclusion in the ontology, therefore ensuring the ontology has content that adheres to its scope ( http://www. obofoundry.org/principles/fp-005- delineated-content.html)	• Review 100 behaviour change intervention evaluation reports • List all entities relevant to the ontology topic found in each report • Select reports that feature a range of health behaviours • HBCP selects reports from a database of reports previously annotated for behaviour change techniques, mechanisms of action and modes of delivery ( Carey *et al.*, 2019)	• 100 reports generate a good initial range of entities for inclusion in the ontology • Using reports targeting a variety of behaviours leads to a greater range of entities being identified than focusing on a single behaviour
b) Re-use entities from existing ontologies appropriately	To respect the principle of inter- operability with existing ontologies ( http://www.obofoundry.org/ principles/fp-010-collaboration. html) and prevent the proliferation of ontology classes with very similar, but not interchangeable, meanings in different ontologies	• Search for terms that have been identified as within scope in existing ontologies, via specialist ontology databases such as the Ontology Lookup Service and BioPortal • Where there are multiple candidate entities from other ontologies that could be re-used, prioritise: a) Ontologies that are actively being maintained b) Ontologies with international relevance c) Entities with clear definitions that capture the meaning required for the new ontology d) Entities whose definitions fit with the intended parent class • Keep track of sources of entities (i.e. record the URI for each one) and follow the Minimum Information to Reference an External Ontology Term (MIREOT) guidance ( Courtot *et al.*, 2011)	• Actively maintained ontologies are more likely to reflect current scientific consensus • Ontologies developed specifically for application in a given country may not work as well applied to other countries • Ontological principles state that the subclass must inherit all the characteristics of the parent class. • Reuse with correct URIs facilitates inter-operability between ontologies
c) Where no suitable entity from an existing ontology identified, search for relevant entities in other classification systems, such as taxonomies and terminologies		• Search sources such as the National Cancer Institute Thesaurus, https://ncit.nci.nih. gov/ncitbrowser/), MeSH terms (Medical Subject Headings, https://meshb.nlm.nih. gov/search) and the International Family of Classifications maintained by the UN Expert Group on International Statistical Classifications ( https://unstats.un.org/unsd/ classifications/Family/ListByDomain • Prioritise classification systems intended to have international relevance • Follow guidance on writing good ontological definitions ( Michie *et al.*, 2019; Seppälä *et al.*, 2017) when converting terms from other classification systems into ontology entities	• To incorporate classification systems widely used in biomedical research • Classification systems developed for national purposes may work less well outside their country of origin
**Step 3: Refining the ontology through an iterative process of literature annotation, discussion and revision**			
a) Apply ontology to annotating research reports	Provides a data-driven basis for selecting and refining entities in the ontology, therefore ensuring the ontology has content that adheres to its scope ( http://www. obofoundry.org/principles/fp-005- delineated-content.html) To examine how terms are used by experts in order to understand the entities in the world being referred to, and then to adjust the ontology and add terms so that the ontology better follows the structure of the world.	• Identify reports to annotate from reports included in high quality systematic reviews of RCTs of behaviour change interventions • For the HBCP, began with RCTs of smoking cessation interventions • Two behaviour scientists use EPPI-Reviewer software. EPPI-Reviewer is web-based software that enables researchers to tag text in pdfs with specific codes relating to the ontology • Create an annotation guidance manual • Keep annotation issues log	• Starting with a behaviour that’s an important public health issue. For the HBCP, smoking cessation is the initial use case • EPPI-Reviewer can automatically produce reports comparing the two researchers’ coding • Annotation guidance manual provides specific guidance on how to annotate reports using the ontology • Due to high variation in how information is reported in reports, there are often uncertainties in annotation. By logging these, they can inform the development of the ontology
b) Regularly reconcile annotating and identify issues leading to discrepancies and revise coding guidance/ ontology		• EPPI Reviewer software can produce a coding summary comparing the annotations of the two researchers • After every 10-15 reports • Update annotation issues log with any issues with ontology term labels or definitions that seem to be leading to differences in coding between researchers	• 10-15 reports found to generate a manageable number of issues for discussion • Log ensures all issues considered systematically. Also enables the identification of recurrent issues with the ontology which may need particular attention
c) Repeat steps a and b		• Annotate another 45 reports in batches of 10-15	• After annotating another 45 reports, few new issues were being noted with the setting or population ontologies
d) Annotate reports relating to a different behaviour and reconcile coding		Selected behaviour should be: • Of importance to human health and wellbeing • The target of numerous behaviour change interventions For the HBCP ontology development process, physical activity is the selected behaviour	• To help ensure ontology is applicable to a wider range of behaviour change interventions than would be the case focusing on only one behaviour
e) Revise ontology		• Based on reconciliation results and annotation issues log after applying the ontology to a different behaviour, consider whether any entities need adding or modifying • If additions required, repeat steps for searching for classes from other ontologies/ classification systems to reuse • Follow guidance on writing ontological definitions when adding or revising entities	• To modify the ontology so that it is applicable to a broader range of behaviour change interventions
**Step 4: Expert stakeholder review**			
	To ensure that the ontology has content that is both scientifically sound and meets the needs of the scientific community ( http://www. obofoundry.org/principles/fp-010- collaboration.html)	• Relevant experts identified through three sources: 1) People who had provided feedback on previous projects at University College London’s Centre for Behaviour Change 2) Authors of reports from less-commonly represented countries, identified through a database of reports using a taxonomy of behaviour change techniques ( www.bct-taxonomy.com/ interventions) 3) People who expressed interest in being involved in Human Behaviour-Change Project expert review tasks in response to invitations on Twitter or in the project newsletter • Invite experts to participate in an online feedback exercise using Qualtrics online survey software • Ask open-ended and closed questions to check: • Relevance – does the ontology include the aspects of its topic that experts see as most important to understanding the effectiveness of behaviour change interventions? • The completeness and clarity of the entities in each section of the ontology. • Members of the ontology development team decide how to respond to each piece of expert feedback, consulting an ontology expert as needed • The ontology is revised accordingly • Team produces document summarising expert feedback with rationale for actions taken in response, shared on the relevant section of Project’s Open Science Framework (OSF) page	• Consulting experts from less commonly represented countries increases the ontology’s global relevance • Inviting stakeholders to self-nominate potentially enhances the diversity of the group • Use of online data collection increases convenience for the experts, maximising response rates and facilitating participation of experts from diverse locations • Sharing responses to the experts’ feedback on OSF increases transparency, demonstrating that each piece of feedback has been systematically considered
**Step 5: Test inter-rater reliability and revise**			
	To ensure that the textual definitions in the ontology provide a human-readable understanding about what is a member of the relevant class ( http://www. obofoundry.org/principles/fp-006- textual-definitions.html)	• First, two researchers leading the ontology’s development annotate 50 intervention reports • Relevant data is extracted from the EPPI- Reviewer “coding records” of the two researchers and the inter-rater reliability coefficient, in the form of Krippendorff’s alpha, calculated • Any systematic discrepancies in coding are identified and the annotating guidance updated if need be • Two behaviour change experts who are new to using the ontology then code 50 reports and inter-rater reliability is calculated as above	• Coding 50 sources will give a 10-15% margin of verror around the estimated percentage agreement between coders ( Gwet, 2014) • Krippendorff’s alpha corrects better for chance than Cohen’s kappa and can be used with multiple data types (nominal, ordinal or interval) ( Gwet, ♠c2014) • Using annotators new to the ontology for final inter-rater reliability testing checks that raters outside the ontology development team will be able to apply the labels, definitions and annotating guidance consistently
**Step 6: Specify relationships between entities in the ontology**			
	Facilitates interoperability between multiple ontologies, especially with respect to logical inference ( http:// www.obofoundry.org/principles/fp- 007-relations.html)	• Use terms from the Relations Ontology	• Required for OBO Foundry registration.
**Step 7: Disseminate and maintain the ontology**			
a) Encode ontology in in Web Ontology Language (OWL)	The ontology should be made available in a common formal language to allow the maximum number of people to access and reuse the ontology ( http://www. obofoundry.org/principles/fp-002- format.html)	• A version of the ontology is stored as an Excel file, containing each entity’s identity, label, definition, relationship with other entities, examples and synonyms • The Excel file is converted into a Web Ontology Language (OWL) file via a series of steps using a tool called ROBOT ( http://robot.obolibrary.org/)	• The Excel file can be prepared by researchers who do not have expertise in generating OWL syntax
b) Publish ontology and report in line with MIRO guidelines		• Make the most recent version of ontology available for download on GitHub • Ensure publications are open access
c) Implement ontology sustainability and change management plan	The ontology needs to reflect changes in scientific consensus to remain accurate over time. ( http:// www.obofoundry.org/principles/fp- 016-maintenance.html)	• Create an ontology issue tracker on GitHub so that any interested party can submit suggestions for additions or improvements to the ontology • Have a named individual who is responsible for moderating change management discussions and seeing the ontology is updated accordingly	• To have an open, sustainable and low-cost portal for the scientific community to suggest and discuss potential changes, which is not reliant on a particular institutional or individual website

### Step 1: Development of the scope and definition of the ontology

***Rationale for step*.** To establish the subject matter the ontology is intended to cover, so ensuring that the ontology only includes relevant content ( http://www.obofoundry.org/principles/fp-005-delineated-content.html). Having a clear overall definition for the ontology makes it easier for future users to identify the ontology as relevant to their interests. ****

***Activities*.** Researchers sought definitions of the overall topic of the ontology from relevant sources, such as scoping reviews of how the entity had been operationalised, previous expert consensus work or, in the absence of such sources, dictionaries. These candidate definitions were discussed within the development team and the overall definition agreed upon.

### Step 2: Identifying key entities and developing their preliminary definitions

***Rationale for step*.** To begin to identify the types of entities the ontology should include. Also, to explore what content relevant to the ontology’s scope might already exist in other ontologies and could be re-used. Developers are encouraged to re-use entities from existing ontologies, where appropriate, to enhance interoperability between ontologies ( http://www.obofoundry.org/principles/fp-010-collaboration.html). This avoids duplication of effort and prevents the proliferation of ontology classes with very similar, but not interchangeable, meanings in different ontologies.

***Activities***

**Data-driven scoping of key entities**

The first activity was data-driven scoping of key entities to include in the ontology. A total of 100 reports were reviewed to identify key entities related to the ontology topic. The reports were randomly selected from a database of interventions targeting a wide range of health behaviours, which were previously coded for behaviour change techniques, mechanisms of action and modes of delivery ( Carey *et al.*, 2019; de Bruin *et al.*, 2016). Using reports that target a variety of behaviours led to a greater range of entities being identified than focusing on a single behaviour. Reviewing 100 reports generated a good initial range of entities for inclusion in the ontology.

**Re-use of entities from existing ontologies and classification systems**

Second, in line with the principle of interoperability with existing ontologies and avoiding ‘re-inventing the wheel’, team members searched for relevant entities from existing ontologies that could be re-used in the new ontology. The search terms were informed by the entities identified through reviewing the 100 reports. Existing ontologies were searched via specialist ontology databases, such as the Ontology Lookup Service and BioPortal. Where entities relevant to the new ontology were identified, their labels and definitions were recorded together with their ***URI***s (unique resource identifiers – a string of characters that unambiguously identifies a particular resource), and the URI of the ontology from which they originated ( Courtot *et al.*, 2011).

Where there were multiple candidates for entities from other ontologies that could be reused, the development team prioritised (a) ontologies that were actively maintained, because they were more likely to reflect current scientific thinking; (b) entities from ontologies that had been developed to have international relevance; and (c) entities with clear definitions that captured the meaning required for re-use within the new ontology. For example, searching the Ontology Lookup Service for “hospital” resulted in four different entities from different ontologies, all with this label, differing in that some focused on a “hospital” as a building where healthcare interventions were delivered, whereas others defined “hospital” as an organisation that delivered healthcare. Therefore, an ontology developer needs to decide whether the entity of “hospital” required for the ontology refers to a building or to an organisation and select an entity to reuse accordingly. In hierarchically structured ontologies, subclasses must share all the characteristics of their parent classes. Therefore, some entities from existing ontologies had to be ruled out as potential additions to the ontology under development because their definitions did not fit with their intended parent class. For example, having decided that the desired parent class for “hospital” was some type of building, the Intervention Setting Ontology development team ( Norris *et al.*, 2020b) ruled out re-using entities that defined “hospital” as a type of organisation.

There were instances where several classification resources that were not ontologies, such as terminologies, contained entities or groups of entities that could be incorporated into the ontology (e.g. the National Cancer Institute Thesaurus. In such cases, the most relevant ones were adapted for use in the new ontology. National classification schemes often worked less well when applied to annotating reports from countries other than were the scheme was developed. For example, both the USA and UK have census categories for ethnic group/”race” but the same words are used to mean different groups – for example, people classified as “Asian” in the UK have different cultural and national backgrounds to those considered “Asian” in the USA. Granularity, or the scale or level of detail, was another important criterion when choosing between competing classification systems to incorporate into the ontology. Classification systems from which the categories could be easily mapped to intervention descriptions in study reports, rather than being too detailed or too broad, were more useful.

For some key entities, no definition was found in an existing ontology or terminology. In such cases, the development team checked whether there was a dictionary definition that could be used. The team only wrote a new definition if they could not find one that characterised the entity they wished to add appropriately for use in an ontology. 

There are a number of principles for “good” definitions of entities in ontologies ( Michie *et al.*, 2019; Seppälä *et al.*, 2017). As a rule, ontological definitions should follow the format: “a [parent class] that [specification of characteristics that set the entity apart from other members of the parent class]” and be intelligible to domain experts. The parent class should be the next highest class in the ontology hierarchy, as this communicates the maximum possible information about the nature of the entity. For example, it is more informative to know that a “hospice facility” is a type of “healthcare facility” (its parent class in the intervention setting ontology) than to know that a “hospice facility” is a type of “intervention site”. The parent class used in a definition should be a single class and not a combination of classes, so the first part of the definition should not use ‘and’ or ‘or’. For example, a library facility should not be defined as a “community facility or educational facility where…” 

Definitions should uniquely identify all the things that belong to the class being defined, and exclude all the entities not in that class, through the careful specification of characteristics that qualify things as members of the class. Therefore, definitions should not simply be lists of the things intended to be included in a class, as these do not help the reader understand what is meant to be included in the class unless they are familiar with those things. For example, defining “Belief about consequences of behaviour” as “Belief such as outcome expectancy or response efficacy” would not enable people unfamiliar with outcome expectancy or response efficacy to understand the key characteristics of this class. Definitions should also avoid simply using a term that has the same meaning as the class’s label, for example defining “addiction” as “dependence on something”. Ideally, ontological definitions avoid using negations (saying what the class is not) unless this is necessary for linguistic clarity or a class is inherently negative. For example, a “child” is better defined as “a human being aged less than 18 years” than as “a human being who is not an adult.” A definition should not include another definition nested within it. If there is a term used in the definition that itself needs defining, ontology developers should create another entry for that term in the ontology. For example, in order to define a library as a type of community facility, developers may also need to create a separate entity for community facility with an appropriate definition. Step 2’s combination of data-driven scoping of entities to include, identifying suitable entities from existing ontologies to re-use, and creating new definitions for entities where needed, resulted in an initial prototype version of the ontology.

### Step 3: Refining the ontology through an iterative process of literature annotation, discussion and revision

***Rationale*.** To ensure the ontology has content that adheres to its scope ( http://www.obofoundry.org/principles/fp-005-delineated-content.html), using a data-driven method for selecting and refining which entities to include in the ontology. Second, to begin to explore whether the textual definitions for classes in the ontology are appropriate, enabling people to understand what qualifies as a member of each ( Seppälä *et al.*, 2017). Third, to examine how terms are used by experts in order to understand the reality being referred to, and then to adjust the ontology and add terms so that the ontology better follows the structure of reality.

***Activities*.** Two behavioural scientists from the ontology development team annotated, or tagged, pieces of text in pdfs of reports with specific codes relating to entities in the ontology using web-based ***EPPI-Reviewer*** software v4 ( Thomas *et al.*, 2010). For example, the phrase “44.5 years” might be annotated as characterising the average age of participants in the study. The team created an ***annotation guidance manual*** for each ontology (e.g. setting https://osf.io/76jty/; mode of delivery https://osf.io/4j2xh/), which provided instructions on how to decide whether particular entities were present in the reports. Since there is considerable variation in how studies are described, often leading to uncertainties as to how best to annotate the different characteristics of a report, the researchers kept an ‘ ***annotation issues’ log*** of uncertainties encountered. The log was also used to note instances of expert authors using a term in a manner that did not seem to fit with the current version of the ontology as well as any relevant intervention or study characteristics found in reports that did not have corresponding ontology classes.

EPPI-Reviewer was used to produce a ***coding record*** comparing the two researchers’ independent annotations for each report. After groups of 10–15 reports had been annotated, the researchers discussed and attempted to resolve any differences, noting any uncertainties about the ontology or annotation guidance. Where annotation difficulties resulted from a paper using a term in a manner that did not seem to fit the current version of the ontology, project team members would discuss each problematic term, trying to understand what aspect of reality was meant by the expert who used the term and then consider how to adjust the ontology to incorporate the entity implied by this usage. Discussion amongst the development team led to revisions to the ontology and/or the annotation guidance manual and to identifying new entities or sub-classes of existing entities. When considering adding new entities to the ontology, the team conducted further searches of existing ontologies and classification systems, using the same methods as in step 2, for relevant entities that could be reused. The revised version of the ontology was used to annotate a further group of 10–15 reports and the ***reconciliation*** and revision process repeated.

To ensure that the ontology had broad relevance to behaviour change interventions, the reports were carefully chosen, ensuring a wide range of intervention types, populations and settings and targeted behaviours of significant public health importance. The first batches of reports focused on smoking cessation interventions and later batches focused on physical activity interventions. The initial focus on smoking cessation was due to its large and relatively well-defined evidence base and having outcome measures that are relatively robust. Physical activity was chosen as the second area because it is a very different behaviour to smoking and easier to measure than diet and alcohol consumption, the two other behaviours of interest to the HBCP. 

The reports annotated were controlled trials of smoking or physical activity interventions selected from two sources: relevant Cochrane Reviews and a database of behaviour change intervention reports whose key features were previously coded for other studies ( Carey *et al.*, 2019; de Bruin *et al.*, 2016). In total 75 smoking cessation reports and 40 physical activity reports were annotated for the Setting and Population ontologies. This quantity was both feasible to annotate but large enough to provide a reasonable basis for ontology refinement. This iterative process of annotating carefully selected literature, discussing and revising the ontology resulted in a version of the ontology with improved coverage of its intended scope and clearer textual definitions for classes.

### Step 4: Expert stakeholder review

***Rationale*.** To review the ontology to ensure it meets the needs of the scientific community and reflects scientific consensus more widely than just within the ontology development team ( http://www.obofoundry.org/principles/fp-010-collaboration.html).

***Activities*.** To maximise response rates and facilitate the participation of experts from diverse locations, expert review was conducted online using Qualtrics software. Relevant expert stakeholders were identified through three sources: (1) people who had provided feedback on previous projects at University College London’s Centre for Behaviour Change, (2) authors of reports from less-commonly represented countries, identified through a database of reports using a taxonomy of behaviour change techniques and (3) people who expressed interest in being involved in HBCP expert review tasks in response to invitations on Twitter or in the project newsletter. Authors of intervention evaluations conducted in less commonly represented countries were consulted to maximise the ontology’s global relevance, given that many of the annotated reports originated from a relatively small group of countries (e.g. USA, Australia, Canada, UK and the Netherlands). Inviting experts to self-nominate via social media is intended to enhance the diversity of the expert group. 

The online survey used a combination of open-ended and closed questions to check:

a) Relevance - whether the ontology covers key elements of a domain that are of interest to the members of the scientific community
b) Representativeness - the completeness of the ontology
c) The clarity of the entities’ labels and definitions from the perspective of domain experts not involved in the ontology’s development

To check coverage of the ontology, experts were asked to identify the characteristics of the topic of the ontology (e.g. “intervention settings”) of interest to them when trying to understand variation in the effectiveness of behaviour change interventions. To make this task more concrete, experts were asked to select a specific behaviour when answering the question, e.g. “eating a healthy diet”. The responses to this question were checked against the areas covered by the ontology to ensure all aspects of the topic considered important by experts had been included.

In the second part of the task, experts were asked to provide feedback on the completeness and comprehensiveness of the ontology. They were presented with each section of the ontology in turn and requested to indicate whether there were (a) any entities missing and, if so, which should be added, (b) any entities, labels or definitions that should be changed and, if so, what changes should be made and (c) any entities that should be placed in a different part of the ontology. Each suggestion was considered by the development team, leading to entities being added to the ontology, edits to labels or definitions or, in some cases, no action (e.g. if an expert suggested adding an entity that was already present in the ontology). To maintain transparency, a log was produced of the team’s responses to each anonymised stakeholder comment. The log for each ontology is publicly available on that ontology’s section of the HBCP Open Science Framework (OSF) page ( https://osf.io/efp4x/). 

### Step 5: Testing inter-rater reliability and making revisions

***Rationale*.** To ensure the clarity of ontology entities’ labels and definitions ( http://www.obofoundry.org/principles/fp-006-textual-definitions.html), by assessing if different people interpret them the same way when annotating reports. Calculating an ***inter-rater reliability*** coefficient provides a benchmark by which to judge whether labels and definitions are sufficiently clear or require revision.

***Activities*.** Inter-rater reliability (IRR) was measured in two stages, first between two researchers leading the ontology’s development and secondly by two behaviour change experts unfamiliar with the ontology but with experience in annotating reports. The latter provided more generalisable knowledge about the extent to which future ontology users are likely to be able to apply the labels, definitions and annotating guidance consistently. The reports annotated for the first IRR assessment were a randomly generated subset of 50, previously unseen, reports taken from a larger dataset of 400 smoking cessation and physical activity reports. Coding 50 reports gives a 10–15% margin of error around the estimated percentage agreement between coders ( Gwet, 2014).

Reliability was measured using Krippendorff’s alpha coefficient ( Hayes & Krippendorff, 2007), which assessed how often researchers disagreed that an entity was present in a report. Krippendorff’s alpha was selected because it can generalise to multiple types of data and any number of coders ( Gwet, 2014; Krippendorff, 2004). Relevant data were extracted from the EPPI-Reviewer “ ***coding records***” of the two researchers and alpha calculated using an automated process developed by the HBCP team ( https://github.com/HumanBehaviourChangeProject/Automation-InterRater-Reliability, version 1.0.0) and incorporating the python script krippendorff 0.3.2 ( https://pypi.org/project/krippendorff/). If the calculated alpha value was less than 0.67 ( Krippendorff, 2009) for the first round of IRR, the reasons underpinning discrepancies in coding were identified and any necessary refinements to the ontology and annotation manual made. 

The reports annotated for the second IRR assessment by experts unfamiliar with the ontology were a random sample of 50 randomised controlled trials from a database of reports coded using a taxonomy of behaviour change techniques, with no restrictions on the outcome behaviour. To maximise the generalisability of the IRR assessment, we selected reports at random from the database ( Gwet, 2014). Again, if the results of IRR testing suggested refinements to the ontology or annotation manual were required, these were made.

### Step 6: Specifying relationships between entities in the ontology

***Rationale*.** To describe the relationships between entities precisely, using terms from the Relation Ontology ( Smith *et al.*, 2005), which was developed to standardise the description of relationships between entities across a wide range of biomedical ontologies. Using standardised terms to describe the relationships between the various entities in the ontology makes it easier to perform computational reasoning both within and across ontologies ( http://www.obofoundry.org/principles/fp-007-relations.html). Describing relationships using Relation Ontology terms is a prerequisite for registering an ontology with the OBO Foundry.

***Activities*.** Common terms from the Relation Ontology ( http://www.obofoundry.org/ontology/ro.html) ( Smith *et al.*, 2005) were used to describe the relationships between entities. In contrast to taxonomies, which are strictly hierarchically structured, a greater range of relationships are possible between the different entities in an ontology. Relation Ontology terms used in the BCIO included the basic hierarchical relationship ‘ *is_a’,* which holds between entities where one is a subclass of another, and ‘has_part’, where a whole has another entity as one of its parts. If ontology developers feel it makes sense to declare a new relation term as part of the ontology itself, this is permissible but the developers are asked to coordinate with the Relation Ontology, for example submitting the new type of relation to the Relation Ontology’s issue tracker ( http://www.obofoundry.org/principles/fp-007-relations.html).

### Step 7: Disseminating and maintaining the ontology

***Rationale*.** To reflect developments and growth in knowledge and the evidence base, associated changes in the scientific consensus and suggestions from the wider scientific community, update the ontology regularly http://www.obofoundry.org/principles/fp-016-maintenance.html). It is important to have a method for collecting and discussing feedback on the ontology, as well as for tracking changes and different versions of the ontology. Ontologies should be disseminated in both human- and computer-readable formats ( Smith *et al.*, 2007), and be freely available for use by all ( http://www.obofoundry.org/principles/fp-001-open.html). The OBO Foundry “common format” principle ( http://obofoundry.org/principles/fp-002-format.html) requires ontologies to be published in an accepted concrete syntax, such as the ***Web Ontology Language*** ( ***OWL***) syntax, which is a widely adopted Worldwide Web Consortium (W3C) standard and therefore allows a wide range of different users and applications to access the ontology content.

***Activities*.** The BCIO will be stored on the Human Behaviour-Change Project ***GitHub*** repository. GitHub provides an open, sustainable and low-cost portal for the scientific community to suggest and discuss potential changes, which is not reliant on the continued functioning of a particular institutional or individual website. It includes an ***issue tracker***, allowing feedback to be submitted which can be openly replied to, discussed and, if appropriate, addressed in subsequent releases of the ontology. GitHub also has in-built mechanisms for tracking releases and ***versioning***, which can be applied as the ontology is updated in response to feedback ( http://www.obofoundry.org/principles/fp-004-versioning.html). The BCIO team has a single designated person responsible for communications between the wider scientific community and the BCIO developers, mediating discussions involving ontology maintenance in the light of scientific advance and ensuring that user feedback is addressed in line with OBO Foundry principles ( http://www.obofoundry.org/principles/fp-011-locus-of-authority.html). It is considered important to have a specific person responsible for communication rather than a group so that responsibility for responding is not diffused. 

Once the initial release has been finalised, the BCIO will be submitted to OBO Foundry for registration. An open access ontology report, conforming to the ***Minimum Information for Reporting an Ontology*** ( ***MIRO) guideline*** ( Matentzoglu *et al.*, 2018) will be published for each ontology (e.g. Norris *et al.*, 2020b). To create the OWL file, first a version of the ontology is created as an Excel file containing each entity’s identity, label, definition, relationship with other entities, examples and synonyms. This Excel file is converted, via a series of steps, into an OWL file using a tool called ***ROBOT***, an automated library and command line tool for ontology workflows ( Jackson *et al.*, 2019). During this step, the logical consistency of the ontology in OWL is checked using an OWL reasoning engine. The Excel and OWL files are both made available in the relevant part of the HBCP repository on GitHub, for example the setting ontology is stored here: https://github.com/HumanBehaviourChangeProject/ontologies/tree/master/Setting.

## Discussion

This method for developing ontologies recommends a clear sequence of activities in order to apply principles of good practice in ontology development when constructing ontologies relevant to behaviour change interventions. We have demonstrated the method to be usable in developing ontologies (e.g. Norris *et al.*, 2020b) that can serve as foundations for a wide variety of scientific activities such as evidence synthesis and its automation, and the study of behaviour change. While there is a wealth of literature on some aspects of ontology development ( Arp *et al.*, 2015; Courtot *et al.*, 2016; Noy & McGuiness, 2001), the Behaviour Change Intervention Ontology (BCIO) development method is novel in terms of its use of formal mechanisms for expert stakeholder involvement and for performing the annotation-driven steps to develop and improve the ontology content.

The MIRO guideline for the minimum information required for reporting ontologies ( Matentzoglu *et al.*, 2018) recommends providing clear description of the steps taken to develop an ontology. This paper provides such a description for the ontologies that are being developed to form part of the BCIO. These will include not only Setting but also Population, Engagement, Behaviour, Mode of Delivery, Style of Delivery, Schedule, Source, Intervention Dose and Mechanism of Action. This method can be applied more generally to developing ontologies relevant to the behavioural and social sciences. For ease of remembering the steps in the method, we suggest adopting the mnemonic SELAR3, representing the first six steps as Scope, Entities, Literature Annotation, Review, Reliability and Relationships.

### Strengths and limitations

The method reported here has been elaborated and the practical recommendations refined through its application to creating two ontologies (Setting and Population). The steps are intended to reflect the principles of good practice in ontology development ( Table 3).

**Table 3.** Steps in the Behaviour Change Intervention Ontology (BCIO) development method related to principles in ontology development.

Principles of good practice for ontologies	Relevant steps in the BCIO development method
1. Having specified scope and content that is scientifically sound and adheres to that scope http://www. obofoundry.org/principles/fp-005-delineated-content. html.	Step 1: defining the scope of the topic, preferably through published expert consensus or a literature review Step 2a: initial review of 100 reports to identify entities relevant to the scope of the ontology Step 3: literature annotation serves to identify further relevant entities
2. Meeting the needs of the relevant community of users http://www.obofoundry.org/principles/fp-010- collaboration.html	Step 4: expert review includes asking which aspects of the ontology topic experts consider important and ensuring these are covered.
3. Following naming conventions, e.g. keeping class names short and memorable, but precise enough to capture the intended meaning ( Schober *et al.*, 2009) and providing appropriate textual definitions for classes, enabling people to understand what qualifies as a member of each ( Seppälä *et al.*, 2017).	Whenever adding or revising ontology entities: follow guidance on writing textual definitions for ontologies Step 3b: comparing the two annotators’ coding identifies problematic labels and unclear definitions that required revision Step 4: expert review seeks feedback on labels and unclear definitions Step 5: inter-rater reliability testing assesses whether two researchers interpret labels and textual definitions similarly.
4. Being logically consistent and having a clear structures, preferably a well-organised hierarchical structure ( Rudnicki *et al.*, 2016).	Use of Basic Formal Ontology to structure the upper levels of the Behaviour Change Intervention Ontology Usually asserting a single parent for each class, rather than multiple parents, creating a clear hierarchy Checking the consistency of the ontology in OWL using an OWL reasoning engine.
5. Maximising the new ontology’s interoperability with existing ontologies by reusing entities from existing ontologies where appropriate http://www.obofoundry. org/principles/fp-010-collaboration.html.	Step 2: searching ontology databases such as the Ontology Lookup Service and BioPortal for entities that could be reused Step 3e: searching these databases again if literature annotation identifies entities that should be added to the ontology
6. The ontology needs to reflect changes in scientific consensus to remain accurate over time. http://www. obofoundry.org/principles/fp-016-maintenance.html	Step 7: the ontology is released with an issue tracker on GitHub, so that members of the scientific community can suggest updates and changes as new research evidence becomes available

One concern is that, generally in ontology development, ensuring ontology terms are understandable by experts from a single domain may not be sufficient to create a clear, organised and scalable vocabulary that can be understood by experts in neighbouring fields or other groups of users. However, the subject matter of the Behaviour Change Intervention Ontology, namely behaviour change interventions and their evaluation, is inherently multidisciplinary, drawing on contributions from psychology, sociology, anthropology, economics, medicine, epidemiology and statistics to name but a few. The literature being annotated to inform ontology development is similarly multidisciplinary. Therefore, the team developing the ontology are forced to avoid the bias of a single perspective on the domain. There are some caveats to the recommended activities that form part of the method. Our estimates of the number of reports needed for different steps in the ontology development process are likely to be conditioned by the nature of the ontology topics; if other groups are developing ontologies to cover broader topics, they may require more reports. Annotation issues logs can give an indication of the future, remaining uncertainties and hence the likely number of further annotated reports needed to resolve them. The success of this data-driven element of ontology development requires judicious selection of reports to ensure a good range of ontology topic characteristics. For example, using reports identified from a systematic review of school-based behaviour change interventions would limit the range of setting characteristics likely to be observed, preventing ontology comprehensiveness. 

There is considerable variation and often ambiguity in information contained in reports. Where descriptions of key intervention characteristics are ambiguous, lower IRR is likely to ensue, even if the definitions and coding guidance for the ontology are clear. Therefore, IRR is only an indicative index of the performance of the ontology’s labels and definitions. Selection of reports for IRR testing should include reports with a wide range of characteristics relevant to the ontology, enabling inter-rater reliability to be tested across the breadth of the ontology.

### Implications for future research and practice

This ontology development method, intended to maximise interoperability of the BCIO with existing OBO Foundry ontologies, increases the potential for future data integration and knowledge accumulation across databases annotated using different ontologies. The method results in ontologies that can be incorporated into computer systems, such as the Knowledge System being developed by HBCP. This will speed up accumulation of knowledge about behaviour change interventions and provide up-to-date knowledge at scale to answer user queries. Each ontology within the BCIO developed using this method can also be applied to improve intervention reporting and evidence synthesis. If other behavioural or social scientists wish to adopt this ontology development method, we recommend they collaborate with others with appropriate expertise to undertake the more technical aspects of the process, such as publishing the ontology in a recognised concrete syntax.

## Conclusions

This ontology development method provides a transparent and systematic approach to developing ontologies relevant to behaviour change interventions, based on accepted principles of good practice. Its use in the successful development of ontologies for setting ( Norris *et al.*, 2020b) and population demonstrates that it is feasible and produces ontologies that have good coverage of the topic area with clear, well-defined entities and have strong potential to meet the needs of the relevant scientific community.

## Ethics

Ethical approval was granted by University College London’s ethics committee (CEHP/2016/555).

## Data availability

### Underlying data

The BCIO is available from: https://github.com/HumanBehaviourChangeProject/ontologies

Archived ontology subsequent to first peer review report : https://doi.org/10.5281/zenodo.3959232 ( Norris *et al.*, 2020a).

License: CC-BY 4.0

### Extended data

Open Science Framework: Human Behaviour-Change Project, https://doi.org/10.17605/OSF.IO/UXWDB ( West *et al.*, 2020).

This project contains the following extended data related to this method:

- Log for each ontology
- Annotation guidance manual

Data are available under the terms of the Creative Commons Attribution 4.0 International license (CC-BY 4.0).

Code used to calculate alpha for IRR: https://github.com/HumanBehaviourChangeProject/Automation-InterRater-Reliability.

Archived code as at time of publication: https://doi.org/10.5281/zenodo.3833816 ( Finnerty & Moore, 2020).

License: GNU General Public License v3.0
