# Supplementary material for: Ontologies relevant to behaviour change interventions: a method for their development
Source: Wellcome Open Res. 2020 Dec 18;5:126. Originally published 2020 Jun 10. [Version 3] doi: 10.12688/wellcomeopenres.15908.3 (PMC7786424; doi:10.12688/wellcomeopenres.15908.3)
Supplement: Peer review history

# Peer review: Reviewer response for version 3

**DOI:** 10.21956/wellcomeopenres.18177.r41884

**Published:** 2021-01-12

**Authors:** Barry Smith, David Limbaugh, Robert Kelly

**Affiliations:** 1 Department of Philosophy, University at Buffalo, Buffalo, NY, USA

1. Introduction

 In our review of Version 1 of Wright et al.’s “Ontologies Relevant to Behaviour Change Interventions: A Method for their Development,” we raised a number of concerns (Kelly et al., 2020). In particular, we focused on the issue of non-conformity with both the upper-level ontology Basic Formal Ontology (BFO) and the realist methodology and principles of best practice underlying BFO (Arp et al., 2015) and adopted by the Open Biological and Biomedical (OBO) Foundry (The OBO Foundry, 2020). Conformity with BFO, the realist methodology, and the associated principles of best practice is critical for building a maximally successful ontology, and Wright and colleagues agree in this new version of their paper. Ultimately, the authors have addressed many of the most fundamental issues we raised in our original review. As the authors note in Version 2 of their paper, many of these were the result of a bug in the OWL code of their ontology that shifted the positions of entities in an input table. The resultant errors have been corrected.

 In the present review of Version 3, we focus on what we take to be some remaining issues with the Behaviour Change Intervention Ontology (BCIO). We are in full agreement with the authors’ endorsement of the principles of best practice identified in Table 3 of their article. In particular, we agree that an ontology should be “logically consistent and having a clear structures [sic], preferably a well-organised hierarchical structure,” and that “Maximising the new ontology’s interoperability with existing ontologies by reusing entities from existing ontologies where appropriate” is critically important (Wright et al., 2020, p. 17). Our remaining concerns with BCIO relate directly to these two principles. First, we identify a number of issues with some of the classifications and definitions in BCIO that seem to be in tension with the just-mentioned principle concerning a well-organized hierarchical structure. Second, we note some reservations about the reuse of certain classes in BCIO, namely from the Gazetteer (GAZ), the Ontology of Medically Related Social Entities (OMRSE), and the Information Artifact Ontology (IAO). While the latter principle of “reuse” is important, it is also important not to let the reuse of existing classes (or their corresponding definitions) compromise the logical integrity or the realist nature of one’s ontology.

 It is worth reiterating that we believe the authors have successfully addressed the most fundamental of the concerns that were raised in the initial reviews. Hence, relatively minor issues notwithstanding, it remains our view that the methods outlined in Wright, et al. (such as the RODM/SELAR3 method) are a valuable contribution to the field, especially the use of formal mechanisms for literature annotation and expert stakeholder review. Moreover, we maintain our

 belief that the Behavior Change Intervention Ontology (BCIO) should and undoubtedly will play an important role in the extension of OBO Foundry ontologies into the behavioral domain.

 The full review can be found here.

Is the rationale for developing the new method (or application) clearly explained?

Yes

Is the description of the method technically sound?

Partly

Are the conclusions about the method and its performance adequately supported by the findings presented in the article?

Partly

If any results are presented, are all the source data underlying the results available to ensure full reproducibility?

Partly

Are sufficient details provided to allow replication of the method development and its use by others?

Partly

Reviewer Expertise:

NA

We confirm that we have read this submission and believe that we have an appropriate level of expertise to confirm that it is of an acceptable scientific standard, however we have significant reservations, as outlined above.

# Peer review: Reviewer response for version 3

**DOI:** 10.21956/wellcomeopenres.18177.r41883

**Published:** 2021-01-05

**Authors:** Emily Arden‐Close

**Affiliations:** 1 Department of Psychology, Bournemouth University, Poole, UK

The authors have addressed my concerns and I am happy to approve the manuscript.

 There is just one minor change: On page 13, paragraph 2, it should read: "countries other than *where* the scheme was developed."

Is the rationale for developing the new method (or application) clearly explained?

Yes

Is the description of the method technically sound?

Yes

Are the conclusions about the method and its performance adequately supported by the findings presented in the article?

Yes

If any results are presented, are all the source data underlying the results available to ensure full reproducibility?

Yes

Are sufficient details provided to allow replication of the method development and its use by others?

Partly

Reviewer Expertise:

Development of health behaviour change interventions. Quality of life in chronic illness

I confirm that I have read this submission and believe that I have an appropriate level of expertise to confirm that it is of an acceptable scientific standard.

# Peer review: Reviewer response for version 3

**DOI:** 10.21956/wellcomeopenres.18177.r41882

**Published:** 2021-01-04

**Authors:** Sofia Strömmer

**Affiliations:** 1 MRC Lifecourse Epidemiology Unit, University of Southampton, Southampton, UK

Thank you to the authors for their considerate response to my comments.

Is the rationale for developing the new method (or application) clearly explained?

Yes

Is the description of the method technically sound?

Yes

Are the conclusions about the method and its performance adequately supported by the findings presented in the article?

Partly

If any results are presented, are all the source data underlying the results available to ensure full reproducibility?

Yes

Are sufficient details provided to allow replication of the method development and its use by others?

Partly

Reviewer Expertise:

Behaviour change interventions, adolescent health, motivation for food choices, health psychology

I confirm that I have read this submission and believe that I have an appropriate level of expertise to confirm that it is of an acceptable scientific standard.

# Peer review: Reviewer response for version 2

**DOI:** 10.21956/wellcomeopenres.17788.r41297

**Published:** 2020-11-23

**Authors:** Emily Arden‐Close

**Affiliations:** 1 Department of Psychology, Bournemouth University, Poole, UK

This is a highly valuable paper. The creation of a method for developing ontologies will, as the authors state, facilitate accurate reporting of behaviour change interventions and synthesis of evidence about effective interventions. Further, the creation of the method follows a thorough, detailed process. It is an honour and pleasure to have the opportunity to read this manuscript and I hope my suggestions will enhance its clarity.

 Figure 1

 This is a complex diagram which was not immediately clear to me. More in-text description of the diagram would be helpful, to show how parts of it link to each other.  I am also not clear why Figure 1 is partly in red and partly in blue. This needs to be explained.

 Page 12 – At the top of page 12, there is a typo – it should read “National classification schemes often worked less well when applied to annotating reports from countries other than where the scheme was developed.”

 Near end of Step 2: “If there is a term used in the definition that itself needs defining, ontology developers should create another entry for that term in the ontology.”  It would be helpful to provide an example here.

 Step 6. If the authors declared any new relation terms, it would be helpful to provide an example here.

Is the rationale for developing the new method (or application) clearly explained?

Yes

Is the description of the method technically sound?

Yes

Are the conclusions about the method and its performance adequately supported by the findings presented in the article?

Yes

If any results are presented, are all the source data underlying the results available to ensure full reproducibility?

Yes

Are sufficient details provided to allow replication of the method development and its use by others?

Partly

Reviewer Expertise:

Development of health behaviour change interventions.               Quality of life in chronic illness

I confirm that I have read this submission and believe that I have an appropriate level of expertise to confirm that it is of an acceptable scientific standard, however I have significant reservations, as outlined above.

# Response

**Published:** 2020-12-09

**Authors:** Alison J Wright

**Affiliations:** UCL, UK

Thank you for taking the time to review our paper and for your constructive feedback.  We will address each comment below.

**Providing greater description of Figure 1**

 As suggested, we have added some additional description of figure 1 to the text of the paragraph immediately following the figure. 

 A fuller description of the upper level of the BCIO is available in the linked upper level paper (Michie et al, 2020) that also forms part of the Human Behaviour-Change Project collection at Wellcome Open Research, and we have now noted this in the paragraph preceding Figure 1.

 Two key entities in the upper level of the BCIO are the Behaviour Change Intervention (BCI) evaluation study and the Behaviour Change Intervention scenario.  Entities relating to the BCI scenario are shown in blue in figure 1. Key BCIO entities relating to the BCI evaluation study are shown in red in figure 1. We have now explained the colour-coding of the figure in the paragraph immediately following it.

**Other issues**

 Thank you for pointing out the typo in the sentence regarding national classification schemes.  This has now been amended.

 As suggested, we have added an example to illustrate the point near the end of Step 2: “If there is a term used in the definition that itself needs defining, ontology developers should create another entry for that term in the ontology.” 

 We did not declare any new relation terms in the Intervention Setting Ontology, so unfortunately cannot provide an example.

# Peer review: Reviewer response for version 2

**DOI:** 10.21956/wellcomeopenres.17788.r40978

**Published:** 2020-11-05

**Authors:** Sofia Strömmer

**Affiliations:** 1 MRC Lifecourse Epidemiology Unit, University of Southampton, Southampton, UK

The creation and refinement of a method for developing ontologies is key to creating shared, scientific terminology to describe health intervention characteristics. Fostering a shared language and understanding will aid reporting and reviewing behaviour change interventions and enable the development of more effective interventions over time. Developing a method for creating ontologies is therefore a very valuable contribution to the scientific community.

 It is an honour to have the opportunity to review this report outlining the development of such a method and I am delighted to contribute to its publication. My review can only be as a potential user of this development method, and of the resulting ontologies in the development and review of health behaviour change interventions. As such, my suggestions are intended to enhance the accessibility of the described method to meet the needs of the relevant community of users.

**Distinction between the BCIO ontology and subclass ontologies within it**

 The paper introduces the Behaviour Change Intervention Ontology (BCIO) and describes it in terms of upper and lower levels. However, the conceptual map in figure 1 requires some dedicated deciphering by the untrained eye as it is not immediately apparent how the hierarchy works. To make this section more accessible, it might be beneficial to shade upper level constructs in the BCIO diagram, and either not shade, or shade with a different colour, the lower level classes. It would also be helpful to highlight the population and setting subclasses in figure 1 as they are used as examples in this paper. If figure one is only the upper level of the ontology, it would be helpful to label this figure as “The Upper Level of the Behaviour Change Intervention Ontology v1.4”.

 It would be useful to clarify the distinction between the BCIO and subclass ontologies. The BCIO seems to be distinct from the development method described in the rest of the paper, which refers to examples of developing ontologies “beneath” nodes of the BCIO. There is a sentence in the discussion (page 15): *“This paper provides such a description for the ontologies that are being developed to form part of the BCIO. These will include not only Setting but also Population, Engagement, Behaviour, Mode of Delivery, Style of Delivery, Schedule, Source, Intervention Dose and Mechanism of Action. This method can be applied more generally to developing ontologies relevant to the behavioural and social sciences.*” This or a similar clarification would be very useful to have at the start of the paper to clearly set out the distinction I refer to above.

 It is the natural tendency for the reader to refer back to figures illustrating a concept when reading about it, in this case the BCIO ontology. It would be good to clarify whether figure one is meant to be considered when reading the guidance that follows it’s introduction, or if the described methods are more relevant for developing the detailed ontology “underneath” some of the nodes in figure 1, e.g. population and context. If so, it would be good to provide an example figure illustrating either those (or part of them if they are too large) so that the reader can follow along and better understand the principles behind creating the ontology. The reason for this suggestion is that on page 12 paragraph 3, principles around subclasses being a specific form of their parent class are outlined, and the example used is that a hospice facility is a type of healthcare facility. The BCIO diagram does not seem to adhere to this rule of thumb. The subclasses in figure 1 are process attributes and parts, not entities that are special forms of their parent nodes. Perhaps this is typical for upper levels of an ontology, if so, it would be helpful to comment on that.  

**Consulting behaviour change experts to refine the ontology (Steps 4 and 5).**

 It is likely that the resulting ontologies will be regularly used by doctoral trainees or early career researchers, for example to conduct systematic reviews. Early career researchers will still be developing their expertise in behaviour change science and may have a different perception on whether labels and definitions are clear and straightforward to use in their work. It would be helpful to comment on this in the paper and perhaps to suggest consulting early career researchers or doctoral students not involved in its development in step 4 or 5 to ensure that the ontology is clear and can be used consistently by behaviour change researchers at different stages of their careers.

 In step 7 you describe the OBO Foundry “common format” principle requiring ontologies to be published in a standardised syntax. Are researchers who set out to develop ontologies expected to become familiar with open source code and tools, or is it more conventional or recommended to collaborate with researchers who have expertise in creating OBO ontologies? Were there specific members of the BCIO team who were dedicated to this task?

 To aid the planning of work for scientists who may be interested in developing ontologies using this method, it would be useful to comment on how long the process of creating the ontologies for *setting* or *mode of delivery* took.

**Minor edits**

 It would be useful to have an example accompanying the following statement on page 11:“Therefore, some entities from existing ontologies had to be ruled out as potential additions to the ontology under development because their definitions did not fit with their intended parent class.“

 Closing parenthesis missing from page 11: “(e.g. the National Cancer Institute Thesaurus.”

Is the rationale for developing the new method (or application) clearly explained?

Yes

Is the description of the method technically sound?

Yes

Are the conclusions about the method and its performance adequately supported by the findings presented in the article?

Partly

If any results are presented, are all the source data underlying the results available to ensure full reproducibility?

Yes

Are sufficient details provided to allow replication of the method development and its use by others?

Partly

Reviewer Expertise:

Behaviour change interventions, adolescent health, motivation for food choices, health psychology

I confirm that I have read this submission and believe that I have an appropriate level of expertise to confirm that it is of an acceptable scientific standard, however I have significant reservations, as outlined above.

# Response

**Published:** 2020-12-09

**Authors:** Alison J Wright

**Affiliations:** UCL, UK

Thank you for taking the time to review our paper and for your constructive feedback.  We will address each comment below.

**Distinction between the BCIO ontology and subclass ontologies within it**

 Thank you for the suggestion that we relabel Figure 1 as only representing just the upper level of the BCIO.  We have implemented this change

 We have now clarified that the detailed methods presented in this paper are those for developing the detail of the ontology “underneath” the classes in Figure 1 to the final paragraph of the section headed “Upper level of the Behaviour Change Intervention Ontology (BCIO) – defining key entities and their scope”

 We have also added a new figure (Figure 2), an extract from the intervention setting ontology that provides an example of some of the lower levels that sit under the upper-level BCIO entity of “behaviour change intervention physical setting.”

 The point regarding subclasses being more specific forms of their parent class applies to writing definitions for ontology classes that have a hierarchical relationship (e.g. “primary school” as a subclass of “school facility”).  However, ontologies are not restricted to having a purely hierarchical structure, which we have now noted in the section on specifying relationships between terms in the ontology (step 6).  Ontology entities can be connected by different types of relationships.  Therefore, processes can have parts (e.g. a behaviour change intervention, which is a process because it is something that occurs over a period of time, has  parts including its content and delivery) and processes can have attributes (e.g. behaviour change intervention delivery can be done in a particular style, or via a particular mode of delivery.)

**Consulting behaviour change experts to refine the ontology (Steps 4 and 5).**

 We agree that early career researchers may have different and valuable perceptions of whether labels and definitions are clear and straightforward to use in their work and so could provide useful feedback on this aspect of the ontology.  However, in step 4, the experts were also asked to provide feedback on the coverage and completeness of the ontology in regard to the field of behaviour change. This requires a greater degree of experience to answer effectively, hence why early career researchers were not consulted at this stage

**Publishing ontologies in an accepted concrete syntax**

 Thank you for inquiring about the technical expertise required to publish the ontology in an accepted concrete syntax, such as OWL.  The BCIO team benefitted from having a team member (JH) who was highly experienced in ontology development, including technical aspects of creating OWL files.  We would recommend that researchers with less technical expertise work with collaborators who can support them with this aspect of the ontology development process.  We have mentioned this in the section on implications for future research.  

**Commenting on how long the process of creating the ontologies for setting or mode of delivery took**

 We agree that information about the time required to develop an ontology might be valuable to other scientists in planning their work.  However, work on both mode of delivery and setting ontologies started before funding had been obtained for the research project, and so proceeded slowly at first.  Also, in both cases, the length of the process was increased due to needing to establish the best methods to use at the same time as attempting the ontology development process.  We therefore don’t think that information about the time taken would be helpful to others.

**Minor edits**

 We have added an example to accompany the statement “Therefore, some entities from existing ontologies had to be ruled out as potential additions to the ontology under development because their definitions did not fit with their intended parent class.”

 We have added to closing parenthesis to “(e.g. the National Cancer Institute Thesaurus)”

# Peer review: Reviewer response for version 1

**DOI:** 10.21956/wellcomeopenres.17449.r39004

**Published:** 2020-07-06

**Authors:** Barry Smith, Robert Kelly, David Limbaugh

**Affiliations:** 1 Department of Philosophy, University at Buffalo, Buffalo, NY, USA

**Abstract**

 In “Ontologies Relevant to BCIs: A Method for their Development” Wright, et al. outline a step by step process for building ontologies of behaviour modification – what the authors call the Refined Ontology Developmental Method (RODM) – and demonstrate its use in the development of the Behaviour Change Intervention Ontology (BCIO). RODM is based on the principles of good ontology building used by the Open Biomedical Ontology (OBO) Foundry in addition to those outlined in (Arp, Smith, and Spear 2015). BCIO uses as its top-level ontology Basic Formal Ontology (BFO). The methods outlined in Wright, et al. are a valuable contribution to the field, especially the use of formal mechanisms for literature annotation and expert stakeholder review, and the BCIO will certainly play an important role in the extension of OBO Foundry ontologies into the behavioural domain. We shall concentrate here, however, on problems with the paper as it now stands, problems which arise primarily from a lack of emphasis on the realist methodology underlying BFO and the OBO Foundry.

 The full review can be found here.

Is the rationale for developing the new method (or application) clearly explained?

Yes

Is the description of the method technically sound?

Partly

Are the conclusions about the method and its performance adequately supported by the findings presented in the article?

Partly

If any results are presented, are all the source data underlying the results available to ensure full reproducibility?

Partly

Are sufficient details provided to allow replication of the method development and its use by others?

Partly

Reviewer Expertise:

Robert Kelly: Bioethics, Applied Ontology Barry Smith: Ontology, Biomedical Informatics, and David Limbaugh's: Applied Ontology, Biomedical Ethics

We confirm that we have read this submission and believe that we have an appropriate level of expertise to state that we do not consider it to be of an acceptable scientific standard, for reasons outlined above.

## References

[1] : Building Ontologies with Basic Formal Ontology. *The MIT Press*.2015; Reference source

[2] : Ontological realism: A methodology for coordinated evolution of scientific ontologies. *Appl Ontol*.2010;5(3-4) : 10.3233/AO-2010-0079 139-188 10.3233/AO-2010-0079 21637730PMC3104413

[3] : A Systematic Analysis of Term Reuse and Term Overlap across Biomedical Ontologies. *Semant Web*.2017;8(6) : 10.3233/sw-160238 853-871 10.3233/sw-160238 28819351PMC5555235

[4] : Pragmatism A New Name for Some Old Ways of Thinking. *Auckland:The Floating Press*.1907;

[5] Information Technology – Top-Level Ontologies (TLO) – Part 2: Basic Formal Ontology (BFO). *International Organization for Standardization*.2020; Reference source

# Response

**Published:** 2020-07-28

**Authors:** Alison J Wright

**Affiliations:** UCL, UK

We would like to thank Smith, Kelly and Limbaugh for their thoughtful review of our paper.

 In section 2.3 of their peer review report, they raise a number of issues regarding potential lack of conformance of the upper-level of the Behaviour Change Intervention Ontology with Basic Formal Ontology. 

 These concerns referred to incorrect relationships that were included in the last released version of the upper level Behaviour Change Intervention Ontology OWL code (bcio_upper_level.owl) due to a bug in the release process (the bug caused the relations to be shifted by one position in an input table, and thus they were assigned the wrong labels in the built version).

 We apologise for this error.  It has now been corrected and the corrected version released on GitHub so that any new accesses will see the right relations.  We hope that this will address the reviewers’ concerns regarding conformance with Basic Formal Ontology.

 We will respond to the other points the reviewers raise in their report soon but wished to correct the bug in the released version of the upper-level Behaviour Change Intervention Ontology as soon as we became aware of it.

# Response

**Published:** 2020-12-09

**Authors:** Alison J Wright

**Affiliations:** UCL, UK

Thank you again for taking the time to read our paper and for your extensive and constructive feedback. We have addressed your comments as follows:

**1.1** **Realist methodology**

 Thank you for the suggestion that the paper would benefit from greater emphasis on the realist methodology underlying BFO and the OBO Foundry. We have added additional text referring to this in the Introduction section paragraph beginning “Ontologies have been developed for numerous scientific domains…”

**1.1.1 Unification**

 We have now briefly mentioned the role of ontologies in providing an anchor for debates that may arise in scientific research by ensuring that all parties at least agree on the real-world referents of the entities they disagree about, even if they disagree about the properties that those entities may hold, also in the paragraph beginning “Ontologies have been developed for numerous scientific domains…”

**1.1.2 Scientifically sound**

 We note the concern that ensuring ontology terms are understandable by experts may be insufficient to create a clear, organized, scalable and interoperable vocabulary that can also be understood by experts in neighbouring fields.  However, the subject matter of this ontology, namely behaviour change interventions and their evaluation, is inherently multidisciplinary, drawing on contributions from psychology, sociology, anthropology, economics, medicine, epidemiology and clinical statistics to name but a few. The literature being annotated to inform ontology development is similarly multidisciplinary.  Therefore, we are forced to avoid the biases of a single perspective on the domain. We have noted this in the paragraph in the Discussion section beginning, “One concern is that…”

 Thank you for the suggestion that it would be beneficial to pair our data-driven approach to building and revising ontologies with a process of carefully examining how terms are used by experts in order to understand the reality being referred to and adjusting the ontology accordingly.  Such a careful examination does take place during the ontology development process, but we realized that we had not made this explicit in the paper.  In particular, during the literature annotation stage, difficulties encountered in coding often resulted from a paper’s expert authors using a term in a manner that did not seem to fit with the current version of the ontology.  During meetings to reconcile coding and revise the ontology, project team members would carefully discuss each problematic term, trying to understand what was meant by the expert who used it, and then debate how the ontology should be adjusted or added to.    We have now revised the text in the section on Step 3 of the method (“Refining the ontology through an iterative process of literature annotation, discussion and revision”) to reflect this, and updated table 2 accordingly.

**2.1 Definitions of key terms**

 We agree that it is useful if all relevant terms are defined precisely.  We are also mindful of the role of the glossary to enable readers, such as many behavioural scientists, who are unfamiliar with the field of ontologies to grasp the meaning of the paper. 

*Artificial intelligence*

 We have revised the definition of artificial intelligence in the glossary to define AI as an occurrent, namely “the practice of building computer programs...” 

 We have also revised the sentence that was highlighted as suggesting that AI is also to be understood as an algorithm, instead now reading, “Algorithms within the knowledge system, developed using artificial intelligence approaches…”

*Interoperability*

 We agree that the definition of “interoperability” would benefit from some revision. The specific concept that we want to help our readers make sense of is “interoperability of ontologies”.  We have therefore updated the definitions table with the reviewers’ suggested definition of “interoperability” and added the further elucidation that “An ontology is interoperable with another ontology if it can be used together with or re-uses parts from the other ontology.”

**2.2 Ontologies as knowledge graphs**

 Thank you for pointing out the need for caution when describing ontologies as representing knowledge.  We have revised the abstract to read “Ontologies are standardised frameworks…” rather than “ontologies are knowledge structures”.  

 The sentence in the introduction previously reading “ontologies encapsulate knowledge” has been refined to read “Because ontologies define key entities in a field…”

**3. Conclusions**

 Thank you for noting that our approach subscribed to many core principles of good ontology design.  We have now mentioned these principles explicitly in the Introduction section on “Best practices in ontology development.”
